# A spectrum of tumorigenesis in an *IDH1*-mosaic patient with Ollier disease, acute myeloid leukaemia, and parathyroid adenoma

**DOI:** 10.3389/fgene.2026.1889201

**Published:** 2026-10-09

**Authors:** Victoria Lopez Delhoulle, Adrien De Voeght, Rafael Fernandez Carazo, Iulia Nechifor-Potorac, Edith Sepulchre, Evelyne Willems, Patrick Pétrossians, Benjamin Koopmansch, Elisa Docampo, Vincent Bours, Saskia Bulk, Frédéric Lambert

**Affiliations:** 1 Department of Human Genetics, CHU Liège, University of Liège, Liège, Belgium; 2 Department of Clinical Haematology, CHU Liège, University of Liège, Liège, Belgium; 3 Department of Endocrinology, CHU Liège, University of Liège, Liège, Belgium

**Keywords:** acute myeloid leukaemia, D-2-hydroxyglutarate, enchondromatosis, *IDH1* mosaicism, mosaicism, Ollier disease, parathyroid adenoma

## Abstract

We report the case of an 18-year-old woman with a childhood diagnosis of Ollier disease—a rare skeletal dysplasia marked by multiple enchondromas and a high risk of malignant transformation into chondrosarcoma. She later developed acute myeloid leukaemia (AML) and a parathyroid adenoma, where the same pathogenic *IDH1* variant [c.394C>T (p.Arg132Cys)] was identified in an enchondroma, AML cells, and the parathyroid adenoma. This gain-of-function variant leads to aberrant production of D-2-hydroxyglutarate, which promotes tumorigenesis through mechanisms such as DNA hypermethylation and impaired cellular differentiation. The presence of this variant in neoplasms arising from both mesodermal tissues (bone marrow, enchondromas) and a predominantly endodermal tissue (parathyroid), with the variant allele frequencies ranging from 37% to 44%, suggests an early postzygotic mosaic event. This case illustrates the broad oncogenic potential of *IDH1* mosaicism and underscores the importance of long-term multidisciplinary surveillance. In particular, the risks of chondrosarcoma and glioma that were previously reported in association with *IDH1-*related Ollier disease support the need for regular surveillance of enchondromas and brain imaging in affected individuals. This case represents the third reported incidence of Ollier disease associated with AML and the first reported incident demonstrating the same *IDH1* (p.Arg132Cys) variant across enchondroma, AML, and parathyroid adenoma, further supporting a broader tumour spectrum associated with *IDH1* mosaicism.

## Introduction

The *IDH1* gene encodes the cytosolic and peroxisomal enzyme isocitrate dehydrogenase 1, which normally catalyses the oxidative decarboxylation of isocitrate to α-ketoglutarate (αKG) and generates NADPH to provide reducing power for cellular antioxidant systems. Somatic oncogenic variants in *IDH1*, predominantly affecting codon 132, have been recurrently identified across multiple tumour types (Tate et al., 2019). These variants confer neomorphic enzymatic activity, enabling the variant enzyme to reduce αKG to D-2-hydroxyglutarate (D-2HG) rather than generate αKG (Liu et al., 2023; Tate et al., 2019). D-2HG acts as an oncometabolite that promotes tumorigenesis by inhibiting αKG-dependent dioxygenases, leading to epigenetic dysregulation and impaired cellular differentiation. It may also modulate the tumour microenvironment by affecting growth factor signalling and antitumour immune responses. D-2HG is excreted in urine as D-2-hydroxyglutaric acid (Liu et al., 2023).

Pathogenic somatic *IDH1* variants are well-established oncogenic drivers in diverse malignancies, including gliomas, chondrosarcoma, cholangiocarcinoma, and acute myeloid leukaemia (AML) (Liu et al., 2023; Yan et al., 2009; Wouters, 2021). When these alterations occur during early embryogenesis, they can lead to somatic mosaicism that manifests as Ollier disease or Maffucci syndrome depending on the lesion type and distribution. Ollier disease is a non-hereditary skeletal dysplasia characterised by multiple enchondromas and a high risk (up to 50%) of malignant transformation into chondrosarcoma (Orphanet, 2025). It is most commonly associated with postzygotic variants in *IDH1* or *IDH2*, although rare alterations involving *PTH1R* have also been reported (Orphanet, 2025). Maffucci syndrome presents similarly but includes soft-tissue haemangiomas. Both conditions have been associated with gliomas, juvenile granulosa cell tumours (JGCTs), and haematological malignancies (Corvino et al., 2024; Michaeli et al., 2025; El Abiad et al., 2020). Herein, we report the third documented case of Ollier disease associated with AML and the second case in which a parathyroid adenoma was observed additionally. In this patient, the same somatic *IDH1* variant [c.394C>T (p.Arg132Cys)] was identified in an enchondroma, AML cells, and the parathyroid adenoma.

## Case presentation

An 18-year-old woman presented with fatigue and pancytopenia in December 2022, whose blood tests revealed anaemia (haemoglobin: 8.6 g/dL), thrombocytopenia (platelet count: 31 × 10^9^/L), leukopenia (white blood cell count: 0.73 × 10^9^/L), and 14% circulating blasts. Bone marrow aspiration showed 76% blasts, and immunophenotyping was consistent with AML (CD45^+^, HLA-DR^+^, CD34^+^, CD117^+^, weak CD33 expression, CD11c, CD13, CD123, partial CD7 expression, and positive myeloperoxidase and lysozyme). Cytogenetics revealed a complex karyotype (49, XX, –7, +8, +8, +9, +21), with fluorescence *in situ* hybridisation indicating *MYC* and *RUNX1* copy-number gains. This cytogenetic profile was classified as adverse risk according to the 2022 European LeukemiaNet criteria. Targeted next-generation sequencing helped identify five pathogenic or likely pathogenic variants as follows: NM_005896.4(*IDH1*):c.394C>T (p.Arg132Cys) (variant allele frequency (VAF): 37%); NM_022552.5(*DNMT3A*):c.2645G>A (p.Arg882His) (VAF: 33%); NM_004364.5(*CEBPA*):c.619G>A (p.Gln207*) (VAF: 25%); NM_004364.5(*CEBPA*):c.230del (p.Phe77Serfs*83) (VAF: 6%); NM_004456.5(*EZH2*):c.1876G>A (p.Val626Met) (VAF: 50%). The classification and supporting evidence for all identified variants are summarised in Supplementary Table S1.

The patient had a prior diagnosis of Ollier disease that was established in 2016 following investigation of bone pain; the radiographs demonstrated multiple enchondromas, and the diagnosis was subsequently supported by histopathological examination of two surgically resected symptomatic lesions. During childhood, she was monitored by a paediatric orthopaedic surgeon annually using radiographic surveillance. After transition to adult care in 2022, she attended a single consultation with an adult orthopaedic surgeon but did not receive further orthopaedic follow-up. Before referral to our genetics clinic in 2025, she had received neither whole-body magnetic resonance imaging (MRI) nor brain MRI examination. She was born at term after an uncomplicated pregnancy and had normal developmental milestones, with no developmental delays or learning difficulties. A physical examination revealed no dysmorphic features apart from short stature (156 cm), which was consistent with the familial stature of her mother and sisters (154–156 cm). Her family history included a father who died from lung carcinoma at 60 years of age in the context of heavy tobacco use; a paternal grandfather with a history of tobacco and alcohol exposure who died from oropharyngeal cancer in his fifties; and a maternal grandmother who developed breast cancer at 40 years of age, contralateral breast cancer at 50 years, and pancreatic cancer at 70 years.

To investigate a potential germline predisposition syndrome, we performed whole-exome sequencing (WES) on DNA from cultured skin fibroblasts; this constitutional tissue selection was based on the fact that peripheral blood obtained after allogeneic haematopoietic stem cell transplantation (HSCT) is unsuitable for germline analysis owing to donor-derived haematopoiesis. Ultra-deep sequencing was not performed on the fibroblast DNA. The detailed methodological information, including sequencing depth, assay-specific limits of detection, and results for each tissue analysed, is provided in Supplementary Table S2. For clinical interpretation, the analysis was restricted to a 96-gene panel associated with inherited myelodysplastic syndrome/AML predisposition and related haematological disorders, including genes associated with inherited bone marrow failure syndromes, covering the corresponding coding regions and canonical splice sites. No pathogenic or likely pathogenic variants were identified. Array comparative genomic hybridisation did not show any pathogenic copy-number variants, except for a 189-kb duplication at 5q23.3 involving CHSY3 (variant of uncertain significance). Chromosome breakage testing with mitomycin C was negative and did not support a diagnosis of Fanconi anaemia or a related chromosome-instability disorder.

The patient achieved complete remission after standard induction chemotherapy (7 + 3: daunorubicin/cytarabine), followed by allogeneic HSCT from an unrelated donor. Two treatment-related complications were subsequently noted. The patient developed premature ovarian insufficiency with vasomotor symptoms and amenorrhoea since March 2023, which was treated with hormone replacement. Notably, the ovarian tissue had been cryopreserved prior to consolidation treatment. Possible cardiotoxicity was also noted, with reduced global longitudinal strain despite a preserved ejection fraction. Electrocardiography showed non-specific repolarisation changes; hence, a temporary cardioprotective regimen with beta-blockers and ACE inhibitors was initiated, as recommended by current guidelines (Lyon et al., 2022). During post-HSCT follow-up, while the patient was still hospitalised in a protected environment, a routine biochemical monitoring revealed severe hypercalcaemia requiring bisphosphonate therapy. Further evaluation showed elevated parathyroid hormone level consistent with primary hyperparathyroidism, and a cervical ultrasonography showed a right inferior parathyroid lesion consistent with an adenoma. The adenoma was surgically resected shortly afterward, resulting in normalisation of serum calcium levels. A histopathological examination confirmed a parathyroid adenoma. The patient has since been monitored annually in endocrinology, with no recurrence of hypercalcaemia. A hereditary predisposition gene panel for hyperparathyroidism (*AIP*, *CDKN1B*, *MEN1*, *PRKAR1A*, and *RET*) did not show any pathogenic or likely pathogenic variants.

Molecular analysis of the adenoma revealed the same *IDH1* (p.Arg132Cys) variant (VAF: 44%) as in the AML. This variant was also identified in a resected enchondroma (VAF: 41%) but was not detected in DNA extracted from cultured skin fibroblasts, supporting somatic mosaicism; however, the absence of the variant in cultured fibroblasts from a single skin biopsy may reflect regional sampling rather than complete absence from fibroblast lineages. Clinically, the patient presented with a right-sided distribution of enchondromas, and the parathyroid adenoma was also located on the right side. We measured D-2-hydroxyglutaric acid level in a spot urine sample for exploratory purposes. While this measurement has not yet been validated for clinical interpretation owing to the absence of studies assessing the stability and longitudinal variability of D-2HG levels over time, it remains of scientific interest. The analysis revealed a total 2-hydroxyglutaric acid concentration of 160 mmol/mol creatinine (368 µmol/L), which is nearly ten times that of the laboratory reference range (6–17 mmol/mol creatinine); of this total, 93.8% corresponded to the D enantiomer and 6.2% was related to the L enantiomer.

## Discussion

Mosaicism arises when a postzygotic genetic or genomic alteration generates two or more genetically distinct cell populations derived from a single zygote. Although considered rare once, mosaicism is now recognised as a common biological phenomenon that reflects the inevitable occurrence of replication errors during the numerous cell divisions required for development and tissue maintenance. The molecular alterations can range from chromosomal abnormalities and copy-number changes to single-nucleotide variants and epigenetic changes, with the phenotypic consequences determined by timing and tissue distribution (Martínez-Glez et al., 2020; Campbell et al., 2015; Waldvogel et al., 2024; Iourov et al., 2019). During early embryogenesis, rapid cell divisions create opportunities for generation and clonal expansion of postzygotic variants. The variants occurring before or around gastrulation (approximately 15–16 d after fertilisation) may contribute to derivatives of more than one germ layer, including ectoderm, mesoderm, and endoderm. The left–right axis specification also begins during early embryogenesis, but the precise temporal relationship between lineage allocation and laterally restricted mosaicism in humans remains uncertain (Campbell et al., 2015; Waldvogel et al., 2024).

In our patient, the *IDH1* (p.Arg132Cys) variant was identified in neoplasms arising from predominantly mesoderm-derived tissues (enchondromas and bone marrow) and from a predominantly endoderm-derived tissue (parathyroid adenoma), with the VAFs ranging from 37% to 44% (Supplementary Table S2). This distribution is consistent with an early postzygotic mosaic event prior to gastrulation. Although the parathyroid epithelium is predominantly endoderm-derived, the neural-crest-derived mesenchyme also contributes to gland development, adding some uncertainty to a strictly germ-layer-based interpretation. Because all the positive results were obtained from neoplastic tissues, the measured VAFs reflect tumour cellularity and clonal architecture and cannot be used to estimate the proportion of variant-positive cells in the corresponding embryonic lineages. The variant was not detected in DNA extracted from cultured dermal fibroblasts; this negative result should be interpreted with caution as it may reflect regional absence of the variant rather than true absence in dermal fibroblasts. This finding is compatible with either a left–right restricted distribution or patchy somatic mosaicism. The predominantly right-sided distribution of the skeletal lesions hints at the possibility that lineage restriction occurred in relation to the left–right axis specification, although patchy expansion of a mosaic clone could produce a similar distribution. Because primordial germ-cell specification occurs during this early developmental period, gonadal involvement and potential transmission cannot be excluded (Campbell et al., 2015; Waldvogel et al., 2024). Figure 1 illustrates a hypothetical developmental model showing one possible timing of the mutational event during early embryogenesis.

**FIGURE 1:**
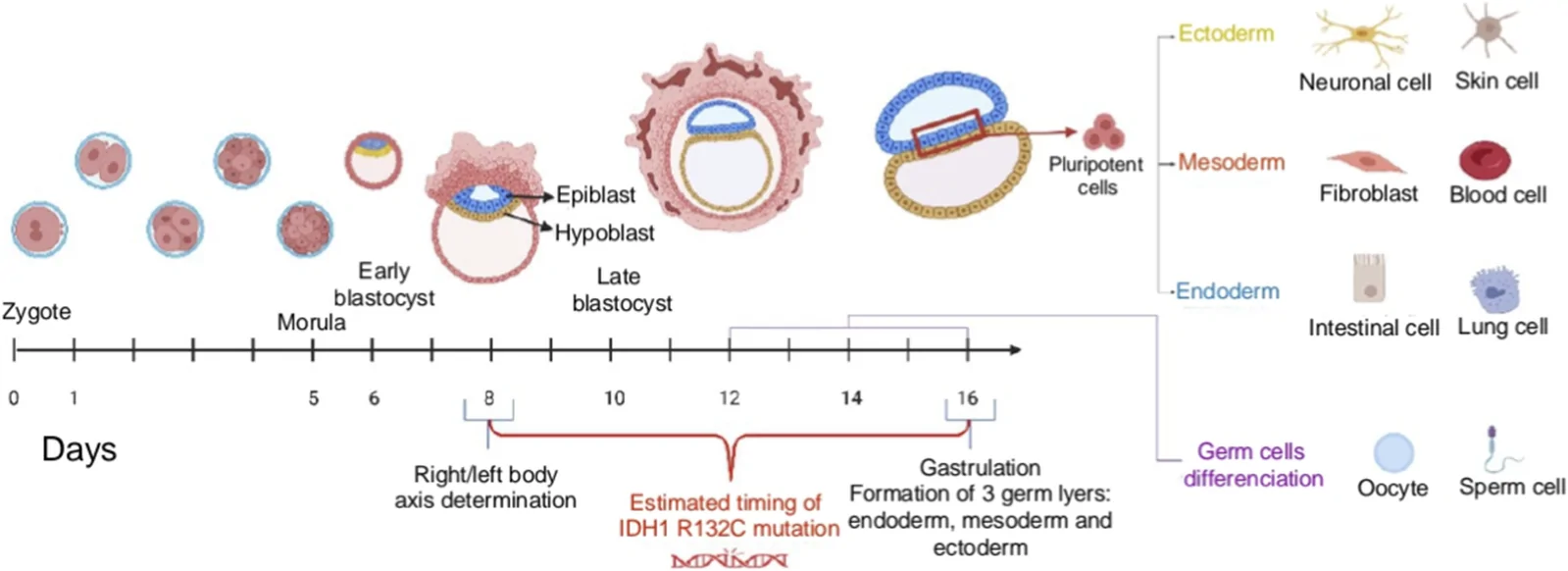
Hypothetical developmental model of the timing of the *IDH1* (p.Arg132Cys) mosaic event in our patient.

Clinically, the germline *IDH1* variants are associated with a distinct developmental syndrome involving severe skeletal, cerebral, and metabolic anomalies; these features were absent in our patient. Furthermore, such germline variants remain exceedingly rare in literature (Illi et al., 2023). The somatic *IDH1* variants are implicated in various cancers: chondrosarcoma (36%–86%), gliomas/glioblastomas (up to 70%), AML (20%), cholangiocarcinoma (5%), and less commonly in thyroid, prostate, or small cell lung cancer (Liu et al., 2023; Yan et al., 2009; Corvino et al., 2024; El Abiad et al., 2020; Mandonnet et al., 2024). These associations support the relevance of *IDH1* variant screening and targeted therapy development (Liu et al., 2023). In our patient, AML harboured additional variants beyond *IDH1*, including *DNMT3A*, *CEBPA*, and *EZH2*, which may represent cooperating leukemogenicity events acquired within the haematopoietic compartment during disease evolution. The *IDH1* variants are considered early events in a subset of myeloid malignancies and may contribute to leukemogenesis through epigenetic dysregulation and impaired cellular differentiation, creating a context permissive for the acquisition of additional alterations (Liu et al., 2023). However, based on a single-timepoint analysis and in the absence of longitudinal pre-leukemic samples, single-cell sequencing, or colony-based genotyping, the precise temporal order of acquisition and clonal hierarchy of these variants cannot be established.

IDH inhibitors are approved for the *IDH1* (p.Arg132Cys) variant in AML (relapsed/refractory or ineligible for standard therapy) and cholangiocarcinoma (second line). However, they are associated with frequent adverse events, such as cytopenia, elevated liver enzymes, QT prolongation, gastrointestinal symptoms, musculoskeletal pain, and peripheral neuropathy. Differentiation syndrome is the most serious consequence of this therapy, requiring urgent administration of corticosteroids and monitoring. A study showed that 99% of patients on *ivosidenib* experienced at least one adverse event, with 21% of the participants experiencing three or more events (Wouters, 2021).

In a cohort of 359 patients (160 Ollier and 199 Maffucci), the median age of the patients with Ollier disease at the time of enchondroma diagnosis was 7 years; of these, 57.8% had unilateral lesions and 53.1% developed malignancies (median age: 25 years). Chondrosarcomas were the most frequent type (≈33%), followed by gliomas (16.1%) and gonadal tumours (8%) (El Abiad et al., 2020). However, these data should be interpreted with caution as Ollier disease is a rare and possibly underdiagnosed condition, and a selection bias is likely given that rare and severe cases are more frequently reported in literature. Our patient shared several features of the typical phenotype of Ollier disease, including childhood onset and unilateral skeletal involvement. However, she developed AML at 18 years of age, which is considerably earlier than the median age of malignancy reported in this cohort, and presented with an additional parathyroid adenoma.

Recently, Luciano et al. (2026) reported the first case of primary hyperparathyroidism caused by an *IDH1* (p.Arg132Leu) variant parathyroid adenoma in a patient with Ollier disease. Similar to our patient, the hypercalcemia was resolved following surgical resection, and both patients developed a parathyroid adenoma at a remarkably young age (24 and 20 years), which is unusual for sporadic primary hyperparathyroidism. While these observations are insufficient to establish a causal relationship, they suggest the possibility that parathyroid adenoma may represent a part of the tumour spectrum associated with *IDH1* mosaicism in Ollier disease rather than a coincidental finding. Notably, three cases of parathyroid adenomas have been reported previously in patients with Maffucci syndrome, although *IDH1/2* variant analyses were not performed in these cases (Luciano et al., 2026). A genetic panel for hereditary hyperparathyroidism in our patient showed no pathogenic variants, further supporting a potential contribution of somatic *IDH1* mosaicism. Although no specific mechanisms have been demonstrated in parathyroid tissue, the oncogenic effects of *IDH1* variants and the associated accumulation of D-2HG are known to promote tumorigenesis in tissues harbouring the variant through epigenetic dysregulation and impaired cellular differentiation.

Previous reports of AML occurring in the context of *IDH1* mosaicism-associated enchondromatosis disorders are summarised in Table 1. To date, only a limited number of cases have been described, including patients with Ollier disease, Maffucci syndrome, and related *IDH1-*associated enchondromatosis phenotypes. Where molecular analysis results were available, recurrent *IDH1* codon 132 variants were identified in AML, with some cases demonstrating the same variant in skeletal and haematological tissues. Corvino et al. (2024) reported an *IDH1* (p.Arg132His) variant shared between enchondroma and AML, while Srinivasan et al. (2020) described an *IDH1* (p.Arg132Cys)-associated AML in a patient with metaphyseal chondromatosis and D-2-hydroxyglutaric aciduria. This spectrum was further expanded in our patient, who demonstrated the same *IDH1* (p.Arg132Cys) variant in enchondromas, AML, and parathyroid adenoma. To further explore the genotype–phenotype correlations, we reviewed published cases on the same mosaic *IDH1* (p.Arg132Cys) variant (Supplementary Table S3). Although these explorations are limited by the small number of molecularly confirmed cases and lack of genetic testing in many historical reports, the available data suggest that the clinical spectrum of *IDH1* (p.Arg132Cys) mosaicism extends well beyond the skeleton (Nejo et al., 2019; Al Rumeh et al., 2020; Srinivasan et al., 2020; Govalan et al., 2021; Littrell et al., 2023; Lv et al., 2022; Zhang et al., 2023; Watanabe et al., 2024; Devins et al., 2024; Amary et al., 2011; Pansuriya et al., 2011; Chen et al., 2022).

**TABLE 1:** Reported cases of *IDH1* mosaicism-associated enchondromatosis and acute myeloid leukaemia (AML).

Reference	Clinical phenotype	Age at AML diagnosis	IDH1 variant	Tissues analysed	Relevance
White et al. (2008)	Ollier disease	7 years	Not reported	Not available	First reported association of Ollier disease and AML
Akiyama et al. (2015)	Maffucci syndrome	7 years	*IDH1* (p.Gly131_Arg132delinsAlaLeu)	AML tissue and haemangioma	Same somatic *IDH1* and *NPM1* (p.Trp288fs) variants identified in haemangioma and AML, supporting a shared clonal origin
Srinivasan et al. (2020)	Metaphyseal chondromatosis with D-2-hydroxyglutaric aciduria	3 years	*IDH1* (p.Arg132Cys)	AML tissue	Supports a continuum of *IDH1*-associated disorders; elevated D-2-hydroxyglutarate with *IDH1*-associated enchondromatosis phenotype
Corvino et al. (2024)	Ollier disease	35 years	*IDH1* (p.Arg132His)	Enchondroma and AML tissue	Same *IDH1* variant identified in cartilage and leukaemia
Present case	Ollier disease	18 years	*IDH1* (p.Arg132Cys)	Enchondroma, AML tissue, parathyroid adenoma	First demonstration of *IDH1* (p.Arg132Cys) variant presence in three neoplastic tissues

The selective tumour spectrum in Ollier disease, rather than a generalised increase in common cancers, may be explained by several factors. The negative selection during embryogenesis might eliminate cells bearing the *IDH1* variant from some tissues. Moreover, tissue-specific *IDH1* expressions and metabolic context may limit D-2HG production, reducing the oncogenic potential. Additionally, genetic modifiers, including unidentified polymorphisms, may influence the tissue-specific vulnerability to *IDH1* variants, as illustrated below by the intronic variant at 8q24.21 linked to increased risk of glioma. Finally, the timing of the postzygotic *IDH1* variant likely shapes its tissue distribution; while the pre-gastrulation variants may involve all germ layers, the later ones may be limited to mesodermal lineages or even cartilage only (Waldvogel et al., 2024; Mandonnet et al., 2024). The risk of glioma warrants particular attention, as the *IDH1* (p.Arg132Cys) variant is a known predisposing factor. Moreover, a germline intronic variant at 8q24.21: NC_000008.11:g.129633446A>G (hg38) transcribed as non-coding RNA (CCDC26) may drastically increase glioma risk when co-occurring with *IDH1* variants by epigenetically dysregulating *MYC* expression (Mandonnet et al., 2024; Yanchus et al., 2022). This variant could not be assessed using the available WES data as this non-coding region was outside the validated scope of clinical interpretation. Thus, dedicated targeted approaches are required for reliable evaluation.

Given the rarity of *IDH1* mosaicism and the absence of evidence-based surveillance guidelines, we propose the following surveillance considerations based on available literature (Michaeli et al., 2025; El Abiad et al., 2020). Whole-body MRI may be performed at diagnosis or as soon as feasible, without general anaesthesia in young children, as the risk of malignant transformation is extremely low during early childhood. After the age of 20–25 years, whole-body MRI may be considered every 1–2 years in adulthood. Neurological surveillance may include a baseline brain MRI at the beginning of adolescence, followed by imaging every 1–2 years to screen for glioma development. Specialised consultations, including comprehensive musculoskeletal and neurological examinations, may be performed every 6–12 months during childhood and every 1–2 years in adulthood. Known enchondromas may be monitored by standard radiographs every 2–3 years, whereas lesions larger than 5–6 cm or located in the pelvis or scapula may warrant annual clinical evaluations and MRI because of their higher reported risk of malignant transformation (Michaeli et al., 2025; El Abiad et al., 2020). Clinical vigilance for symptoms suggestive of gliomas (neurological symptoms) or ovarian JGCTs (lower abdominal pain) is also justified (Michaeli et al., 2025; El Abiad et al., 2020). Given the possibility of gonadal mosaicism, genetic counselling should be offered and preimplantation genetic testing must be discussed with patients considering future pregnancy (Martínez-Glez et al., 2020; Campbell et al., 2015; Iourov et al., 2019).

The therapeutic perspectives include emerging interest in the use of IDH inhibitors to treat enchondromas by reducing D-2HG levels with the aim of alleviating pain, improving bone mineralisation, and potentially reducing the risk of malignant transformation, as recently reported by Funck-Brentano et al. (2024). However, the use of such inhibitors remains controversial owing to high costs and the significant side-effect profile. Urinary D-2-hydroxyglutaric acid level has also been proposed as a potential biomarker, although its clinical utility remains unvalidated. In our patient, the D-2-hydroxyglutaric acid level measurement was performed for exploratory purposes only and was based on a single post-HSCT urine sample (El Abiad et al., 2020).

## Conclusion

This case illustrates the tumour spectrum associated with *IDH1* mosaicism, including Ollier disease, AML, and parathyroid adenoma. It underscores the need for specialised referral centres combining expertise in rare bone disorders, oncology, and genetics to manage such complex presentations. It also emphasises the urgency of establishing international registries to better characterise the phenotypic spectrum of *IDH1* mosaicism and optimise evidence-based surveillance protocols.

## Data Availability

The original contributions presented in the study are included in the article/Supplementary Material; further inquiries can be directed to the corresponding author.
