# Supplementary material for: A spectrum of tumorigenesis in an IDH1-mosaic patient with Ollier disease, acute myeloid leukaemia, and parathyroid adenoma
Source: Front Genet. 2026 Oct 9;17:1889201. doi: 10.3389/fgene.2026.1889201 (PMC13690521; doi:10.3389/fgene.2026.1889201)
Supplement: Supplementary file 1 [file DataSheet1.pdf]

Supplementary

Supplementary Table S1. Classification and interpretation of the genetic variants identified in the present case.

**Variant**	**Classification**	**Somatic variant interpretation criteria (ComPerMed/AMP-ASCO-CAP framework)**	**ACMG classification criteria**
IDH1; c.394C>T; p.(Arg132Cys)	Pathogenic	Oncogenic hotspot, ComPerMed Consensus Pathogenic Variants list (CPV), tumour recurrence (>500 COSMIC entries), Pathogenic in ClinVar, deleterious missense prediction tools; REVEL score: 0.814.	PM1_Moderate; PM2_Moderate; PP3_supporting; PP5_Supporting
DNMT3A; c.2645G>A; p.(Arg882His)	Pathogenic	Oncogenic hotspot, ComPerMed Consensus Pathogenic Variants list (CPV), tumour recurrence (>1000 COSMIC entries), Pathogenic in ClinVar, deleterious missense prediction tools; REVEL score: 0.742.	
CEBPA; c.619C>T; p.(Gln207*)	Likely pathogenic	Clear loss-of-function (LOF) variant: premature stop codon in Tumour Suppressor Gene	
CEBPA; c.230del; p.(Phe77Serfs*83)	Likely pathogenic	Clear loss-of-function (LOF) variant: frameshift and premature stop codon in Tumour Suppressor Gene	
EZH2; c.1876G>A; p.(Val626Met)	Likely pathogenic	Tumour recurrence (>10 COSMIC entries); Pathogenic in ClinVar; deleterious missense prediction tools; REVEL score: 0.817.	

Supplementary table S2: Methodological characteristics and molecular results for the detection of the *IDH1* c.394C>T; p.(Arg132Cys) variant across the different tissues analysed.

**Sample**	**Molecular assay**	**Depth at *IDH1* c.394C>T**	**Limit of detection (LOD)**	**Variant allele frequency (VAF)**	**Notes**
Bone marrow at AML diagnosis	Targeted NGS (AML gene panel; SOPHiA Genetics), MiSeq (Illumina)	3,411×	2%	37%	Diagnostic AML sample
Enchondroma 1	ddPCR (*IDH1* c.394C>T Variant Detection Assay)	Not available	1%	41%	
Enchondroma 2	ddPCR (*IDH1* c.394C>T Variant Detection Assay)	Not available	1%	Inconclusive	Insufficient DNA for reliable analysis
Parathyroid adenoma	Targeted NGS (thyroid gene panel; SOPHiA Genetics), MiSeq (Illumina)	5,487×	6%	44%	
Skin biopsy DNA	Targeted NGS (HaloPlex panel; Agilent), MiSeq (Illumina)	7,420×	5%	3%	Low-level variant signal below the validated limit of detection; not considered a confirmed positive result
Cultured skin fibroblasts	Whole-exome sequencing (SureSelect Human All Exon v6; Agilent), NovaSeq (Illumina)	Not available	30–50%	Not detected	Fibroblast culture removes contaminating blood cells that may be present in the original skin biopsy, which may explain the absence of the low-level signal observed in biopsy DNA. Whole-exome sequencing is not designed to detect low-level mosaic variants
Peripheral blood after HSCT	Not analysed				Not suitable for constitutional testing because haematopoiesis was donor-derived following allogeneic HSCT

Table S3. Clinical spectrum of patients with mosaic *IDH1* p.Arg132Cys variant

**Nejo et al. (2019)**	Male	Maffucci syndrome	Adulthood (age not specified in available data)	Maffucci syndrome with adenoma, diffuse astrocytoma, and chondrosarcoma	*IDH1* p.(Arg132Cys) detected in pituitary adenoma, diffuse astrocytoma and clival chondrosarcoma; blood negative	NR	One of the strongest demonstrations of p.(Arg132Cys) mosaicism affecting multiple independent tissues. Shows that absence of pathogenic variant in blood does not exclude widespread tissue mosaicism.
**Al Rumeh et al. (2020)**	Male	Ollier disease history	Glioma diagnosed at 23 years	Ollier disease associated diffuse astrocytoma	*IDH1* p.(Arg132Cys) detected in astrocytoma tissue	NR	Supports association between p.(Arg132Cys) mosaicism and glioma development in Ollier disease.
**Srinivasan et al. (2020)**	Male	Early childhood metaphyseal chondromatosis with D-2-hydroxyglutaric aciduria	3 years (AML presentation)	*IDH1*-associated enchondromatosis phenotype with D-2-hydroxyglutaric aciduria	*IDH1* p.(Arg132Cys) detected in AML cells (and reported in association with cartilage phenotype)	Elevated	First report linking p.(Arg132Cys) mosaicism with AML and increased D-2HG levels. Highly relevant to present case because of overlap between haematological malignancy and *IDH1*-driven metabolic phenotype.
**Govalan et al. (2021)**	Male	Ollier disease history	29 years	Ollier disease with liver tumour	*IDH1* p.(Arg132Cys) detected in liver tumour tissue	NR	Suggests that p.(Arg132Cys) mosaicism may predispose to rare visceral neoplasms such as liver tumour.
**Littrell et al. (2022)**	Female	Ollier disease during childhood	Juvenile granulosa cell tumour diagnosed at 15 years	Ollier disease associated juvenile granulosa cell tumour	*IDH1* p.(Arg132Cys) detected in ovarian tumour tissue	NR	Further illustrates ovarian tumour susceptibility in patients carrying p.(Arg132Cys) mosaicism.

**Haiyan Lv et al. (2022)**	Male	Maffucci syndrome diagnosed at 19 years	Chondrosarcoma diagnosed at 45 years	Maffucci syndrome with chondrosarcoma	*IDH1* p.(Arg132Cys) detected in chondrosarcoma tissue, absent from blood	NR	Documents tumour-restricted mosaicism and malignant transformation driven by p.(Arg132Cys) variant clones.
**Zhang et al. (2023)**	Female	Ollier disease during childhood	Juvenile granulosa cell tumour diagnosed at 4 years	Ollier disease associated juvenile granulosa cell tumour	*IDH1* p.(Arg132Cys) detected in both ovarian juvenile granulosa cell tumour and enchondroma	NR	Demonstrated identical p.(Arg132Cys) variant in cartilage and ovarian tumour, supporting a common embryonic mosaic clone and expansion beyond skeletal tissues.
**Watanabe et al. (2024)**	Male	Ollier disease diagnosed at 1 year	Multiple gliomas diagnosed at 15 years	Ollier disease with multiple gliomas	*IDH1* p.(Arg132Cys) detected in multiple gliomas	NR	First detailed p.(Arg132Cys) Ollier case with malignant transformation of gliomas. Highlights that the variant may predispose to both cartilage and CNS neoplasms.
**Devins et al. (2024)**	Female	Ollier disease during childhood	Ovarian tumour diagnosed at 10 years	Ollier disease with ovarian sex cord stromal tumour	*IDH1* p.(Arg132Cys) detected in ovarian tumour by NGS	NR	Expands p.(Arg132Cys) associated tumour spectrum to ovarian mesenchymal tumours.
**Amary et al. (2011)**	Cohort: Not individually reported	Not individually reported	Not available	Ollier disease and Maffucci syndrome	Enchondromas ± chondrosarcomas and soft tissue haemangiomas	Increased in tumour tissue in 4 participants	40 subjects (19 with tumour samples available). One subject developed brain tumour.<br>p.(Arg132Cys) represented ~65% of variants with *IDH1* alteration.
**Pansuriya et al. (2011)**	Cohort: Not individually reported	Not individually reported	Not available	Ollier disease (n=43), Maffucci syndrome (n=13)	Enchondromas, chondrosarcomas	NR	*IDH1* p.(Arg132Cys) was the predominant *IDH1* variant.

					and spindle cell haemangiomas		
**Chen et al. (2022)**	Cohort: 5 patients (3 females, 2 males)	Childhood to early adulthood (individual data available but heterogeneous)	Variable	Ollier disease (n = 3) and Maffucci syndrome (n = 2)	Enchondromas, chondrosarcomas, spindle cell haemangiomas, ovarian juvenile granulosa cell tumour, ovarian serous cystadenoma, breast carcinoma†	NR	Single-institution clinicopathological series demonstrating identical *IDH1* p.(Arg132Cys) variants across multiple neoplastic lesions within the same patients, supporting a common post-zygotic mosaic origin and expanding the tumour spectrum beyond cartilage.
**Present case**	Female	Ollier disease diagnosed at 11 years	AML and parathyroid adenoma diagnosed at 18 years	Ollier disease with AML and parathyroid adenoma	*IDH1* p.(Arg132Cys) detected in enchondroma, AML and parathyroid adenoma	Elevated urinary D-2HG	First reported case demonstrating the same *IDH1* p.(Arg132Cys) variant in enchondroma, acute myeloid leukaemia and parathyroid adenoma. This case further expands the recognised tumour spectrum associated with post-zygotic *IDH1* p.(Arg132Cys) mosaicism.
